# Incubation and Transmission of Epidemic Parotitis

**Published:** 1887-08

**Authors:** 


### SELECTIONS.

### Incubation and Transmission of Epidemic Parotitis.

Three cases are reported by Dr. Roth, which aid in establishing the time of incubation and the manner of transmission of mumps: The period of incubation in all three cases was eighteen days. The first case was caused by actual contact; in the second, the infectious material was apparently brought by the physician himself from a patient in the hospital to another patient in his home; in the third case, the patient used the same bedding which had previously been used by a patient with parotitis.—*Boston Medical and Surgical Journal, May* 19, 1887.

